# Mutational Landscapes of Normal Skin and Their Potential Implications in the Development of Skin Cancer: A Comprehensive Narrative Review

**DOI:** 10.3390/jcm13164815

**Published:** 2024-08-15

**Authors:** Tae-Ryong Riew, Yoon-Seob Kim

**Affiliations:** 1 Department of Anatomy, Catholic Neuroscience Institute, College of Medicine, The Catholic University of Korea, Seoul 06591, Republic of Korea; 2 Department of Biomedicine and Health Sciences, College of Medicine, The Catholic University of Korea, Seoul 06591, Republic of Korea; 3 Department of Dermatology, Bucheon St. Mary’s Hospital, College of Medicine, The Catholic University of Korea, Seoul 06591, Republic of Korea

**Keywords:** squamous cell carcinoma, normal skin, genomic, mutation, mutational signature, cancer, driver, copy number alteration, next-generation sequencing

## Abstract

Recent evidence suggests that physiologically normal skin harbors pervasive mutant clones with cancer drivers. Normal skin has the highest burden of somatic mutations due to persistent ultraviolet exposure throughout life. The mutation burden exponentially increases with age and is further modified by skin site, sun-damage history, and skin phototype. Driver gene profiles in normal skin are similar to those in cutaneous squamous cell carcinoma where *NOTCH* family, *FAT* family, and *TP53* are consistently reported, while other reported profiles include *PPM1D*, *KMT2D*, *ASXL1*, and *RBM10*. Normal skin seldom harbors canonical hotspot mutations with therapeutic relevance. The pathologic role of mutant clones with cancer drivers in normal skin is classically considered precursors for skin cancer; however, recent evidence also suggests their putative cancer-protective role. Copy number alterations and other structural variants are rare in normal skin with loss in 9q region encompassing *NOTCH1* being the most common. Study methodologies should be carefully designed to obtain an adequate number of cells for sequencing, and a comparable number of cells and read depth across samples. In conclusion, this review provides mutational landscapes of normal skin and discusses their potential implications in the development of skin cancer, highlighting the role of driver genes in early malignant progression.

## 1. Background

It has been widely accepted that cancers arise as a result of the accumulation of somatic mutations, and that the majority of somatic mutations are present in normal tissues before cancer development [1,2,3,4]. While the mutational landscapes in advanced stage cancers have been comprehensively profiled by large consortia [5,6], our understanding of these in normal tissues is primitive. However, this understanding is quickly expanding due to recent studies investigating various types of normal tissues [7,8,9,10,11,12]. Given the frequent association of positively selected mutant clones with common cancer drivers, mutant clones in normal tissues are considered to be the origin of cancer, providing potential insights into early-stage carcinogenesis [3]. Interestingly, recent evidence suggests that the mutant clones in normal tissues also have potential cancer-protective roles [13] or are involved in the pathogenesis of aging and complex traits [14,15,16]. Detecting somatic mutations in normal tissue is challenging due to the small size of mutant clones and their polyclonal composition [17]. To overcome these limitations, recent next-generation sequencing (NGS) studies have adopted sampling and analysis strategies capable of reliably detecting small mutant clones in an unbiased way [3].

Sun-exposed normal skin is known to contain widespread mutant clones harboring cancer drivers, making it the normal tissue with the highest number of somatic mutations due to persistent ultraviolet (UV) exposure [17,18,19,20,21,22]. Cutaneous squamous cell carcinoma (cSCC) is the second most prevalent form of skin cancer, posing a substantial socioeconomic burden due to rapidly increasing prevalence [23]. Given the common link between driver genes found in normal skin and those in cSCC, it is widely accepted that normal skin, especially chronically sun-exposed skin in elderly individuals, represents the earliest stage of carcinogenesis in cSCC [17,18,19,20,21,22]. Genomic characterization of normal skin will facilitate the identification of biomarkers for early detection and targeted therapies of mutant clones that develop into skin cancer as well as risk stratification for skin cancer development. Moreover, as cSCC typically presents as a spectrum of progressively advanced malignancies ranging from normal skin, actinic keratosis to invasive cSCC, genomic characterization of normal skin will help predict high-risk lesions of actinic keratosis for cSCC progression [24,25]. While other studies have conducted sequencing on bulk tissue of normal skin (primarily keratinocytes) [17,18,19,20,21], single-cell sequencing of melanocytes and fibroblasts also showed widespread somatic mutations and genome alterations due to UV exposure, endogenous DNA damage, and DNA replication errors [22,26,27].

In this review, we aim to comprehensively review the current literature for the mutational landscapes of normal skin. We focused on the current evidence of driver genes, mutational signatures, and copy number alterations (CNAs) in normal skin and discussed the potential implications for the development of skin cancer. We further discuss several special considerations for the genome study of normal skin. Compared to previous reviews for the mutational landscape of normal tissues [1,2,3,4], our review focuses more on results from normal skin, especially on mutational signatures, and CNAs, and their implications for the development of skin cancer.

## 2. Mutational Landscapes of Normal Skin

**Figure 1:**
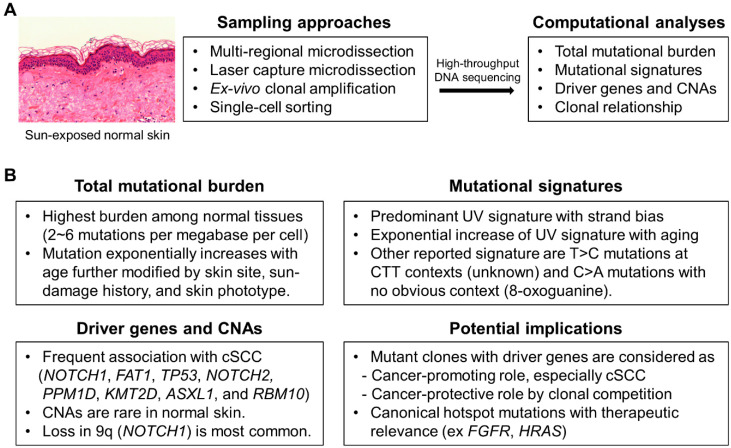
Summary for the study methodologies and the mutational landscapes for genomic studies in normal skin. (**A**) Example histology of sun-exposed normal skin tissue is shown. Sampling approaches and computational analyses are summarized. (**B**) Mutational landscapes of normal skin in terms of total mutational burden, mutational signature, driver genes under positive selection, and copy number alterations (CNAs) with the potential implications were summarized.

Somatic mutations gradually accumulate in normal tissue throughout life by two primary causes: somatic mutations acquired during embryonic development followed by neutral drift, and other mutations occurring in post-mitotic or actively dividing cells as a result of exposure to endogenous or exogenous insults [3]. To detect somatic mutations in a small number of cells within normal tissue, sampling approaches include multi-regional (manual or laser capture-based) microdissection, ex vivo clonal amplification, and single-cell sorting, followed by high-throughput DNA sequencing [1] (Figure 1A). From computational analyses of sequencing data, mutational landscapes of normal skin can be investigated in terms of total mutational burden, driver genes under positive selection, mutational signatures, copy number alterations (CNAs), and clonal relationship between mutant clones (Figure 1A). Mutant clones with cancer drivers have been identified in nearly all normal tissues to different extents, suggesting that positive selection of mutant clones is relatively common [1]. It has been classically known that sun-exposed skin contains clonal patches carrying *TP53* mutations [28,29,30]. Recent NGS-based studies have revealed that sun-exposed skin harbors pervasive mutant clones with cancer drivers and exhibits the greatest number of somatic mutations among normal tissues, probably due to persistent UV exposure [17,18,19,20,21,22]. We have compiled the key findings from notable studies of normal skin (Table 1), and provided an overview of the mutational landscape of normal skin (Figure 1B).

The landmark study analyzing 234 small biopsy samples of normal skin from eyelid skin showed that the burden of somatic mutations is comparable to common cancers and the frequency of cancer drivers is remarkably high in normal skin (2~6 mutations per megabase per cell) [17]. Subsequent studies in larger numbers of normal skin samples consistently showed a high burden of somatic mutations and the frequent detection of cancer drivers in cancer driver genes [18,19,20]. Exponential accumulation of somatic mutations was found in accordance with increasing age, matching skin cancer incidence, and the increase in mutational burden is in turn modified by skin site, sun-damage history, and skin phototype [18,19]. For the majority of mutations in normal skin, variant allele frequencies were typically less than 10%, indicating only a small proportion of cells carrying mutations [17]. Interestingly, multiple cancer genes are under strong positive selection in normal skin, including most of the key drivers of cSCC rather than those of basal cell carcinoma (BCC) and melanoma [17,18,19]. As expected, the most predominant mutational signature in normal skin is UV signature [17,18,19].

While most previous studies have performed sequencing of bulk skin tissue mainly comprising keratinocytes, a recent study investigating the genomic landscape of melanocytes in normal skin at the single-cell level found a distinct mutational landscape: known pathogenic mutations in melanoma and high mutational burden in intermittently sun-exposed skin compared to chronically sun-exposed skin [22]. Mutation burden is higher in melanocytes adjacent to a skin cancer than those from cancer-free donors, and phylogenetic analyses identified groups of clonally related melanocytes in the same skin regions [22]. Taken together, these findings indicate that normal skin harbors individual melanocytes or fields of clonally related melanocytes that contain pathogenic mutations driving melanoma [22].

A prior study of single-cell-derived clonal fibroblast lineages from two healthy adult humans showed that fibroblasts harbored between 600 and 13,000 base substitutions, with mutational signatures of UV-induced and endogenous DNA damage, and at least one chromosomal rearrangement [26]. Consistently, a recent study investigating whole-genome sequencing of single-cell-derived clonal lineages of fibroblasts and melanocytes from healthy individuals (*n* = 21) found widespread genomic changes due to UV exposure, endogenous DNA damage, and DNA replication errors [27]. UV-induced mutations were prominent in many samples, even in sun-shielded skin, and were independent of age. In contrast, spontaneous deamination of methylated cytosines and insertions/deletions (indels) characteristic of DNA replication errors increased linearly with age [27]. Interestingly, UV-induced mutations were more prevalent in Caucasian donors compared to African-American donors, suggesting potential racial differences [27]. Collectively, these findings indicate that normal skin harbors mutant clones not only in epidermal cells but also in fibroblasts.

## 3. Driver Genes under Positive Selection

Driver genes under positive selection can be identified by analyzing significantly mutated genes through the relative degree of the excess of non-synonymous mutations compared to synonymous mutations [31]. Driver genes show high tissue specificity with clear differences across normal tissues [32]. Our previous study of whole-exome sequencing-based unbiased analysis of genes suggested *NOTCH1*, *FAT1*, *TP53*, *PPM1D*, *KMT2D*, and *ASXL1* as driver genes under positive selection in normal skin [33]. From other panel sequencing-based studies of normal skin, *NOTCH1*, *FAT1*, *TP53*, and *NOTCH2* have been consistently identified as driver genes [17,18,19]. Other reported driver genes include *NOTCH3*, *RBM10*, *KMT2D*, and *ASXL1* [17,18,19]. In addition, site-specific driver genes were reported where *TP53* preferentially selected in the head and *FAT1* in the leg [19]. Interestingly, although BCC is the most common skin cancer worldwide, drivers of BCC including the pivotal driver gene of *PTCH1* were rarely mutated in normal skin [17,33]. One of the major differences between normal tissue and cancer is the number of cancer drivers where a normal cell harbors 0.27 driver point mutations per cell compared to 2.7 per tumor in cSCC; however, some clones in normal skin may harbor 2–3 cancer drivers, raising the question of what combinations of events are sufficient for the transformation [17].

Although positive selection of mutant clones with cancer drivers is well-defined in normal skin, their natural progression remains poorly understood. The clonal fitness of mutant clones depends on not only their genotype, but also the dynamic equilibrium with the cellular microenvironment [3,34]. For example, in normal epithelium, clone growth is constrained by the limited size of the proliferating compartment and competition with surrounding cells, indicating the presence of protective mechanisms that maintain epidermal integrity [35]. Consistently, in a study of transgenic lineage tracing in a mouse model of esophageal carcinogenesis, diverse mutant clones undergo clonal competition depending on the mutations they carry and the nature of the neighboring cells, and tissue homeostasis is achieved when clones with similar competitive fitness collide [34]. Interestingly, recent observations suggest that expansion of mutant clones in the lung due to exogenous mutagens of smoking can be reversible after cessation of smoking [11]. In normal skin, positive selection of mutant clones with cancer drivers is strong only during the initial exponential expansion of mutant clones, followed by neutral drift [36]. Accordingly, a study of transgenic mouse epidermis from both skin and esophagus inducing a single-allele p53 mutation (*Trp53* R245W) showed that progenitors with the *Trp53* mutation initially outcompeted wild-type cells, but subsequently reverted toward normal dynamics and homeostasis [37,38].

## 4. Potential Implications of Driver Genes

Mutant clones with cancer drivers in normal skin are classically considered to be precursors of skin cancer. This view is supported by the observation that multiple cancer drivers including most of the key drivers of keratinocyte cancer, especially cSCC, are under strong positive selection in normal skin [17]. Moreover, the number and the size of positively selected clones increase with age and are associated with known cancer risks [18,19]. The same applies to melanocytes in normal skin where known driver genes of malignant melanoma are found to be positively selected in melanocytes at the single-cell level [22]. Some driver genes are more frequently observed in skin cancers than in normal skin, suggesting the importance of acquiring mutations in those driver genes for cancer development. For example, *TP53*, *NOTCH2*, and *KMT2D* mutations are enriched in cSCC compared to normal skin [19]. Normal skin rarely shows *CDKN2A* point mutations, which were frequent drivers in cSCC, suggesting that *CDKN2A* inactivation appears to be specific to cancer clones [17]. In spite of clear signs of positive selection in known cancer driver genes, only a few in normal skin progress into cancer, implying that determinants of clonal expansion are at least partially different from those of cancer transformation [32]. These findings also imply that some frequently mutated genes in cancer may be inherited from the normal progenitors but play no role in cancer transformation [32].

Intriguingly, normal tissue sometimes shows a higher frequency of mutations in certain well-known cancer drivers (e.g., *NOTCH* genes in skin and esophagus) compared to the corresponding cancers [3]. Additionally, the peak age of melanoma patients with mutant *NOTCH1* is delayed compared to those with wild-type *NOTCH1*, suggesting that *NOTCH1* mutations may be protective against melanoma [39] These findings imply that mutant clones with cancer driver mutations might have a cancer-protective role depending on the context and timing of their mutations [3,40]. Indeed, this hypothesis is supported by two pivotal experimental studies on animal models of esophageal carcinogenesis. These studies consistently reported that the expansion of normal cells with a *Notch1* mutation eliminates early neoplasms harboring wild-type *Notch1* through clone competition and extrusion, thereby preserving tissue integrity [13,41]. Since previous studies have primarily investigated esophageal tissues with *Notch1* mutations, further research in various cancer models is required to elucidate the potential cancer-protective role of mutant clones with cancer driver mutations in normal tissues.

## 5. Canonical Hotspot Mutations

Normal skin seldom harbored several known canonical hotspot mutations in oncogenes such as *FGFR3* (K652E, Y375C), *KRAS* (G12V, G12D), *HRAS* (G12D, G13D), and *NRAS* (Q61R) mutations in a previous study [17], and in our previous study, found *HRAS* (Q61L), and *PIK3A* (H1047Y) mutations [33]. Accordingly, another previous study reported canonical hotspot mutations such as *BRAF* (F595L, V600E), *HRAS* (G12D, G12V), *PIK3CA* (P471L, E542K, E726K, M1043I, H1047L), and *FGFR3* (R248C, S249C, Y373C, A391E) [18]. On the other hand, canonical hotspot mutations in melanoma such as mutations in *BRAF* codon 600 residue and *TERT* promoter mutations were also not observed in melanocytes from normal skin [22]. Some of these hotspot mutations in normal skin have proved to be oncogenic with available druggable targets, indicating potential therapeutic relevance [42]. Further studies are needed to elucidate whether targeting these canonical hotspot mutations will be relevant for preventing carcinogenesis in normal skin and why mutant clones with canonical hotspot mutations did not transform into cancer.

## 6. Mutational Signatures

In our previous study, de novo extraction of mutational signatures in normal skin from whole-exome sequencing data (*n* = 39) found a single signature with main components of UV (SBS7a and SBS7b), followed by aging (SBS1 and SBS5) [33]. Most of the mutational signature of normal skin can be explained by the extracted signature for most of the samples, indicating that mutational processes other than UV exposure and aging are less likely to be involved [33]. Consistent with these findings, UV signature with C > T mutations in dipyrimidine sites or CC > TT mutations is found to be prevalent in the mutational process of normal skin from previous studies, although these studies adopted targeted sequencing rather than a genome-wide methodology [17,18,19,20,22]. Furthermore, besides UV signatures, C > T mutations due to spontaneous cytosine deamination (SBS1) are prevalent in fibroblasts of normal skin, and these mutation burdens correlated with age, indicating the possible role of the aging process on mutations in normal skin [27]. Consistent with known transcription-coupled nucleotide excision repair due to UV exposure, C > T and CC > TT mutations were significantly more frequent on the non-transcribed strand compared to the transcribed strand [17]. The decline of nucleotide excision repair capacity with aging was proposed as a possible explanation for the exponential increase of UV signature with aging [18,43].

A previous study reported a signature of T > C mutations at CTT contexts, which was predominantly observed in younger donors under the age of 63 [18]. This mutational signature closely resembled the SBS17a signature, a signature with unknown etiology that has been shown to contribute to cutaneous melanoma [18]. The distinct mutational signature between age groups can potentially be attributed to the exponential increase of UV signature with age, compared with the linear increase in T > C mutations with age [18]. On the other hand, another study reported a signature of C > A mutations without obvious sequence context but with strand bias [17]. This signature was also found in cells after in vitro exposure of cells to UV [44], and observed in cSCCs, but less frequently in BCCs and melanomas [17]. The proposed etiology for this signature is the oxidation of guanine residues by reactive oxygen species, particularly 8-oxoguanine, generated by sunlight [45].

Normal skin may harbor unidentified mutagens, including genetic deficiencies and environmental pollutants. The PCAWG large consortia study showed that the mutational signature of cutaneous melanoma includes APOBEC (SBS2/13) and reactive oxygen species (SBS18), in addition to signatures of UV exposure and aging [46]. Although these signatures were not detected as major mutational signatures in normal skin in previous studies, further research is needed for a definitive conclusion. Furthermore, skin cancers other than melanoma were not included in the PCAWG study and have been investigated in relatively smaller studies. In both in vitro and in vivo experiments, arsenic exposure has been shown to have a synergistic effect with UV, leading to more than a two-fold enrichment of UV-related mutational burden [47]. In a study of human cSCC, mutational signature analysis reveals the presence of a novel signature, the signature related to exposure to the immunosuppressive drug azathioprine [48]. Although results are mixed, some studies have indicated an increased risk of skin cancer in patients with *BRCA1/2* mutations [49]. Further studies are needed to investigate the potential role of mutagens other than UV and aging in normal skin.

## 7. CNAs

While CNA is a pervasive trait of human cancers involving more than 80% of certain types of tumors, CNAs are rarely involved in normal skin. The most frequently reported CNA event in normal skin is CNA in the 9q region encompassing the *NOTCH1* gene, which was identified in 11.5% (27/234) [17], 4.3% (2/46) [19], and 5.1% (2/39) [33] of samples. Interestingly, most *NOTCH1* CNAs co-occurred with *NOTCH1* point mutations in a single sample of normal skin, indicating possible biallelic inactivation [17,33]. Relatively fewer instances of CNAs in normal skin compared to cancer drivers can be explained either by the relatively higher sensitivity of mutation detection compared to CNA detection, or by the possibility that CNAs may occur in a relatively later stage of skin carcinogenesis than cancer drivers. Structural variants other than CNAs such as structural rearrangements and chromosomal abnormalities are also rarely reported in normal skin [3] and other normal tissues [50], although false-negative detection cannot be ruled out due to the relatively lower sensitivity of detecting these types of alterations in small mutant clones. Recent advances in single-cell sequencing and bioinformatics technologies have enabled precise assessment of CNAs at the single-cell level [51,52,53,54]. Sequencing analyses of induced pluripotent stem cell lines derived from primary skin fibroblasts of healthy individuals revealed widespread CNAs in approximately 30% of the fibroblast cells [55]. However, another study reported a rare occurrence of aneuploidy (less than 5%) in human skin, brain, and liver [56]. Most previous studies of CNAs have either relied on generating cell cultures or sequencing single-cell genomes, both of which are inherently error-prone and do not allow for independent validation of mutations. Due to these technical challenges, research on CNAs in normal skin is relatively underdeveloped compared to studies on mutations. Further research is needed to elucidate CNAs in normal skin and explore their potential implications.

## 8. Special Considerations for Genome Study of Normal Skin

Several special considerations need to be considered for genome study of normal skin in methodological and histological aspects. The limitations and potential biases of different study methodologies, such as sample acquisition and sequencing methods, should be acknowledged. Somatic mutations in normal cells generally occur at low frequencies; for example, a constant mutation rate of 13–44 mutations per genome per year has been observed, depending on the tissue type [1]. Consequently, in many normal tissues, the average size of clones is expected to be very small, leading to very low variant allele frequencies. Ideally, unbiased sequencing of single-cell genomes could reliably represent the mutational landscape of normal tissue. However, this approach is challenging due to various artifacts introduced by genome amplification. To overcome these limitations, several methodologies can be used, each with its own potential drawbacks: in vitro culture of single cells can lead to the selection of specific clones and the introduction of culture-related mutations; sequencing of clonal populations, such as a single intestinal crypt, and laser capture microdissection of small regions can directly measure mutations in a clonal population, but may suffer from reduced sensitivity and potential false findings [1]. A sampling strategy needs to be carefully designed to obtain a suitable number of cells (corresponding to the sample size) where the sample size needs to be large enough to obtain a sufficient amount of DNA for appropriate sequencing technology, but small enough to be captured within the sensitivity limitation of mutation detection [1].

Sequencing technologies impact the sensitivity and specificity of detecting small mutant clones in normal tissue. There are three major types of genome sequencing: whole genome sequencing (WGS), whole exome sequencing (WES), and targeted deep sequencing (TDS). WGS and WES provide comprehensive coverage of the entire genome or exome and offer reliable detection of mutational signatures. However, they generally have lower coverage in specific regions and require larger sample amounts and higher sequencing costs. In contrast, TDS focuses on particular clusters of genomic regions, offering the highest read depth and requiring smaller sample amounts and lower sequencing costs. However, TDS may not capture genomic regions outside the targeted panel, and cannot investigate the clonal architecture of mutant clones, and the mutational signatures [57]. Reliable detection of small mutant clones necessitates a higher read depth, but this must be carefully controlled, as a higher read depth can lead to the detection of a relatively higher number of mutations [58].

Mutational landscapes significantly vary across normal tissues, which is likely to result from not only the cellular fitness contributed by somatic mutations due to intrinsic and extrinsic mutagens, but also other histological factors such as natural tissue architecture, tissue proliferation rate, and tissue microenvironment [3,21]. Sampling of the targeted cell type without contamination of unwanted cell types is ideal; for example, contamination with dermal fibroblasts and leukocytes should be avoided for studying keratinocytes. Histological tissue architecture can influence clonal expansion in which the clones typically remain small in size in tissues with a more complex architecture [59]. For example, most mutant clones with cancer drivers are confined to the clonal unit of a single gland in normal tissues such as colon and endometrium [7,8], while mutant clones with cancer drivers are mosaically distributed in normal tissues with planar epithelium without structural restraints, such as skin and esophagus [60]. Due to the absence of a clonal unit in normal skin, tissue size is a crucial factor for the sensitivity of mutation detection, and multi-regional sequencing is needed for the precise inference of positive selection of mutant clones. In addition, histological location is also important as keratinocytes in hair follicles, and those in the interfollicular epidermis, have distinct mutational landscapes [19]. Accordingly, some melanocytes had fewer mutations than other melanocytes in the same biopsy sample of sun-exposed skin, indicating the possible protected niche from sun damage such as in hair follicles [22].

## 9. Conclusions

In this comprehensive review, we examined the current literature on the mutational landscapes of normal skin, focusing on total mutational burden, mutational signatures, driver genes under positive selection, and CNAs, along with their potential implications for the development of skin cancer. We also discussed canonical hotspot mutations and special considerations for genomic studies of normal skin. Our review highlights the putative role of driver genes in the malignant progression from normal skin to skin cancer. As most previous studies on normal skin have utilized targeted deep sequencing of small biopsy specimens mostly composed of keratinocytes, further studies need to be performed with finer resolutions genome-wide and at the single-cell level. Moreover, further integrative analyses with transcriptome and epigenome data and pathological spatial profiles will help us understand molecular alterations in spatial contexts during cancer evolution. For example, the application of emerging techniques such as single-cell and spatial sequencing multi-omics technologies in skin cancer tissue with adjacent normal skin may allow us to precisely characterize the molecular alterations and spatial contexts underlying the early-stage carcinogenesis of skin cancer.

## Figures and Tables

**Table 1:** Main findings from representative studies of normal skin.

Ref (Year)	Patient (*n*)	Study Methods (Sample (*n*))	Main Findings
[17] (2015)	4	TDS of small biopsies of eyelid skin (0.8 to 4.7 mm^2^) (*n* = 234)	Average mutation burden of 2-6/Mb per cell and the major mutational signature of UV Significantly mutated genes of *NOTCH1-3*, *FAT1*, *TP53*, and *RBM10*. Positively selected mutations in 18–32% of normal skin cells: ~140 cancer drivers per cm^2^ *NOTCH1* is the most frequent gene with CNAs (27/234)
[22] (2020)	6	TDS of flow-sorted melanocytes after tissue culture of skin biopsy (*n* = 159)	Mutation burden is higher in intermittently sun-exposed skin than in chronically sun-exposed skin. Mutation burden is higher in melanocytes adjacent to a skin cancer than in cancer-free donors. Melanocytes from normal skin commonly contained pathogenic (weakly oncogenic) mutations. Phylogenetic analyses identified groups of clonally related melanocytes in the same skin regions.
[19] (2021)	35	TDS of small biopsies of normal skin (2 mm^2^) (*n* = 1261)	Most mutations were caused by UV, but there are differences in DNA-repair processes between sites. *TP53* preferentially positively selected in the head and *FAT1* in the leg. Fine-scale mapping revealed 10% of clones had CNAs. Mutations in the upper hair follicle resembled adjacent skin, but the lower follicle did not.
[18] (2021)	123	TDS of small biopsies of normal skin (*n* = 123)	Exponential accumulation of UV-related somatic mutations with age, matching skin cancer incidence. The increase of mutational burden is in turn modified by an individual’s skin phototype. Somatic mutations preferentially accumulated in cSCC cancer genes and clonally expanded with age. Mutational signature analyses suggest a loss of fidelity in transcription-coupled repair later in life.
[20] (2021)	13	TDS of small biopsies of normal skin from postmortem donors (*n* = 450)	Mutation burden and driver genes were significantly different between SE and NE areas. Hotspot mutations in *TP53*, *NOTCH1*, and *GRM3* significantly associated with UV exposure. Mutational counts in cSCC were associated with the UV-induced mutation counts in skin with the difference mostly conferred by the low-frequency mutations.
[27] (2021)		WGS of single-cell derived clonal lineages of fibroblasts and melanocytes (*n* = 21)	Each skin cell carries from 402 to 14,029 base substitutions, 7 to 71 indels and 1 to 14 structural variants per cell including known cancer drivers. UV-induced mutations were prominent in many samples even in sun-shielded skin, and were age-independent. Spontaneous deamination of methylated cytosines and indels characteristic of DNA replication errors were also found with a linear increase with age.
[31] (2023)	39	WES of small biopsies of normal skin (9 mm^2^) (*n* = 39)	Median mutation burden was higher in exposed skin (10.4/Mb) than non-exposed skin (0.25/Mb). Significantly mutated genes of *NOTCH1*, *FAT1*, *TP53*, *PPM1D*, *KMT2D*, and *ASXL1*. Single mutational signature in normal skin with components of UV and aging. Rare instances of copy-neutral loss of heterozygosity in 9q (*n* = 2) and 6q (*n* = 1).

CNA (copy number alterations); cSCC (cutaneous squamous cell carcinoma); NS (normal skin); TDS (targeted deep sequencing); UV (ultraviolet); WES (whole-exome sequencing); WGS (whole-genome sequencing).

## Abbreviations

Basal cell carcinoma (BCC); Copy number alteration (CNA); Cutaneous squamous cell carcinoma (cSCC); Next-generation sequencing (NGS); Ultraviolet (UV).
